# Investigating the Genetic Diversity of H5 Avian Influenza Viruses in the United Kingdom from 2020–2022

**DOI:** 10.1128/spectrum.04776-22

**Published:** 2023-06-26

**Authors:** Alexander M. P. Byrne, Joe James, Benjamin C. Mollett, Stephanie M. Meyer, Thomas Lewis, Magdalena Czepiel, Amanda H. Seekings, Sahar Mahmood, Saumya S. Thomas, Craig S. Ross, Dominic J. F. Byrne, Michael J. McMenamy, Valerie Bailie, Ken Lemon, Rowena D. E. Hansen, Marco Falchieri, Nicola S. Lewis, Scott M. Reid, Ian H. Brown, Ashley C. Banyard

**Affiliations:** a Virology Department, Animal and Plant Health Agency, Addlestone, Surrey, United Kingdom; b WOAH/FAO International Reference Laboratory for Avian Influenza, Swine Influenza and Newcastle Disease, Animal and Plant Health Agency (APHA-Weybridge), Addlestone, Surrey, United Kingdom; c School of Biological Sciences, University of Manchester, Manchester, United Kingdom; d Agri-Food and Bioscience Institute, Belfast, United Kingdom; e Veterinary Exotics and Notifiable Disease Unit, Animal and Plant Health Agency, Addlestone, Surrey, United Kingdom; f Department of Pathology and Population Sciences, Royal Veterinary College, University of London, Hertfordshire, United Kingdom; g Worldwide Influenza Centre, The Francis Crick Institute, London, United Kingdom; Institute of Microbiology, Chinese Academy of Sciences

**Keywords:** WGS, avian influenza, genomics, high-pathogenicity

## Abstract

Since 2020, the United Kingdom and Europe have experienced annual epizootics of high-pathogenicity avian influenza virus (HPAIV). The first epizootic, during the autumn/winter of 2020–2021, involved six H5Nx subtypes, although H5N8 HPAIV dominated in the United Kingdom. While genetic assessments of the H5N8 HPAIVs within the United Kingdom demonstrated relative homogeneity, there was a background of other genotypes circulating at a lower degree with different neuraminidase and internal genes.  Following a small number of detections of H5N1 in wild birds over the summer of 2021, the autumn/winter of 2021–2022 saw another European H5 HPAIV epizootic that dwarfed the prior epizootic. This second epizootic was dominated almost exclusively by H5N1 HPAIV, although six distinct genotypes were defined. We have used genetic analysis to evaluate the emergence of different genotypes and proposed reassortment events that have been observed. The existing data suggest that the H5N1 viruses circulating in Europe during late 2020 continued to circulate in wild birds throughout 2021, with minimal adaptation, but then went on to reassort with AIVs in the wild bird population. We have undertaken an in-depth genetic assessment of H5 HPAIVs detected in the United Kingdom over two winter seasons and demonstrate the utility of in-depth genetic analyses in defining the diversity of H5 HPAIVs circulating in avian species, the potential for zoonotic risk, and whether incidents of lateral spread can be defined over independent incursions of infections from wild birds. This provides key supporting data for mitigation activities.

**IMPORTANCE** High-pathogenicity avian influenza virus (HPAIV) outbreaks devastate avian species across all sectors, having both economic and ecological impacts through mortalities in poultry and wild birds, respectively. These viruses can also represent a significant zoonotic risk. Since 2020, the United Kingdom has experienced two successive outbreaks of H5 HPAIV. While H5N8 HPAIV was predominant during the 2020–2021 outbreak, other H5 subtypes were also detected. The following year, there was a shift in the subtype dominance to H5N1 HPAIV, but multiple H5N1 genotypes were detected. Through the thorough utilization of whole-genome sequencing, it was possible to track and characterize the genetic evolution of these H5 HPAIVs in United Kingdom poultry and wild birds. This enabled us to assess the risk posed by these viruses at the poultry-wild bird and the avian-human interfaces and to investigate the potential lateral spread between infected premises, a key factor in understanding the threat to the commercial sector.

## INTRODUCTION

Since 2020, high-pathogenicity avian influenza virus (HPAIV) outbreaks have devastated the poultry sector globally and constitute a significant challenge to food security. Avian influenza viruses (AIVs) are classified as being of either low-pathogenicity or high-pathogenicity HP (1, 2). Low-pathogenicity AIVs (LPAIVs) generally cause mild infections, while HPAIVs can cause high rates of mortality in a wide range of avian species. AIV subtypes are defined based on their surface glycoproteins, hemagglutinin (HA) (H1 to H16) and neuraminidase (NA) (N1 to N9) (3), but HPAIVs appear restricted to the H5 and H7 subtypes. In many countries, legislation is in place for the statutory control of H5 and H7 AIVs as notifiable animal pathogens (2) under the direction of the competent veterinary authorities (4–6). Commonly, national measures for HPAIV prevention and control focus on stringent biosecurity and the depopulation of affected flocks with compensation to control outbreaks (7). As such, the surveillance and monitoring of wild bird and poultry populations for clinical signs are critical for the detection and rapid control of such outbreaks (2). Wild bird populations can maintain both HPAIVs and LPAIVs, and seasonal migration is considered a key factor in the intercontinental dissemination of these viruses. The mixing of bird species at different sites enables genetic reassortment following coinfection, resulting in the emergence of novel AIVs (8). Where infection pressure is high in birds and/or the environment, there is an increased risk of spread to poultry as well as an increasing interface with other species, including humans (through occupational exposure) and scavenging animals (9–13). However, the basis of species-to-species adaptation events remains undefined.

AIVs are enveloped, negative-sense, single-stranded RNA viruses, with each virion containing 8 genome segments that together can generate up to 22 different core and accessory proteins (14), including the polymerase complex (polymerase basic protein 2 [PB2], PB1, and polymerase acidic protein [PA]), the nucleoprotein (NP), the viral glycoproteins (HA and NA), structural proteins (matrix 1 [M1] and M2), and nonstructural proteins (NS1 and NS2). Critically, the polymerase complex lacks proofreading abilities, so polymerase errors can occur, leading to genetic drift and the subsequent maintenance of errors through successive generations. Ultimately, the polymerase error rate drives the evolution of these viruses, having an estimated error rate of between 10^−4^ and 10^−3^ substitutions per nucleotide (15–19). A further critical factor in the genetic evolution of these viruses is genetic reassortment following coinfection of the same cell. This feature of influenza virus biology can lead to dramatic genetic shifts that can result in the emergence of novel influenza viruses, some with altered characteristics.

During the 2020–2021 autumn/winter season, the United Kingdom of Great Britain and Northern Ireland as well as the British Crown Dependencies (referred to as the United Kingdom hereafter) and Europe experienced a significant AIV epizootic (20), with 5 H5 HPAIV clade 2.3.4.4b subtypes (H5N1, H5N3, H5N4, H5N5, and H5N8) and at least 19 distinct genotypes being observed (21). However, this multisubtype epidemiological scenario changed dramatically during 2021–2022 with the emergence of a dominant H5N1 HPAIV clade 2.3.4.4b subtype, with only a small number of infections due to other subtypes (H5N2 and H5N8) being reported (22). Despite the dominance of the H5N1 subtype, significant genetic diversity was observed within these viruses across Europe. The viruses initially detected in Europe possessed an HA gene with high similarity to that observed in the H5N1 viruses detected during the 2020–2021 epizootic and into the summer of 2021 (23, 24). This H5N1 sublineage, termed the B1 sublineage (23), is ancestral to those viruses that have been detected in North America since late 2021 (25, 26). Later detections of H5N1 during the 2021–2022 epizootic identified a second HA sublineage, B2, which encompassed viruses detected across Europe and demonstrated divergence brought about by the accumulation of amino acid substitutions (23). The divergence observed within the HA gene was accompanied by additional diversity in the other 7 influenza virus gene segments, resulting in a total of 16 genotypes by November 2021 (25).

In this study, we generated whole-genome sequencing (WGS) data for 240 AIVs from wild birds and poultry between 2020 and 2022. We have analyzed outbreak cluster data to assess the possible differentiation between independent incursions and interrogate the potential for lateral spread between infected premises (IPs).

## RESULTS

### Incursions from wild birds drove the rapid emergence of the dominant H5N8 HPAIV subtype during the autumn/winter of 2020–2021.

The detection of HPAIV in Europe in the autumn of 2020 signaled that HPAIV was reemerging (27), with the virus first being detected in the United Kingdom in a greylag goose (Anser anser) in Gloucestershire on 30 October 2020. Wild bird detections during that season were limited to a 14-week period from 30 October 2020 to early February 2021, totaling 311 detections in Great Britain (28), with 9 further detections in Northern Ireland. While multiple H5Nx HPAIV subtypes were detected across Europe, H5N8 dominated wild bird detections in the United Kingdom, with 96% (*n* = 292/320) of detections, while 4% were H5N1 (*n* = 13/320), 2% were H5N5 (*n* = 6/320), and 0.3% were H5N3 (*n* = 1/320). The NAs of the remaining samples were untyped (H5Nx) (3%; *n* = 8/320). Additionally, 26 HPAIV-infected poultry premises were detected in the United Kingdom, beginning with H5N8 HPAIV in Cheshire on 2 November. Twenty-three further H5N8 HPAIV detections and two H5N1 HPAIV detections were made up to 31 March 2021. Two notifiable LPAIV infections (H5N2 and H5N3) were also detected on poultry premises.

**FIG 1:**
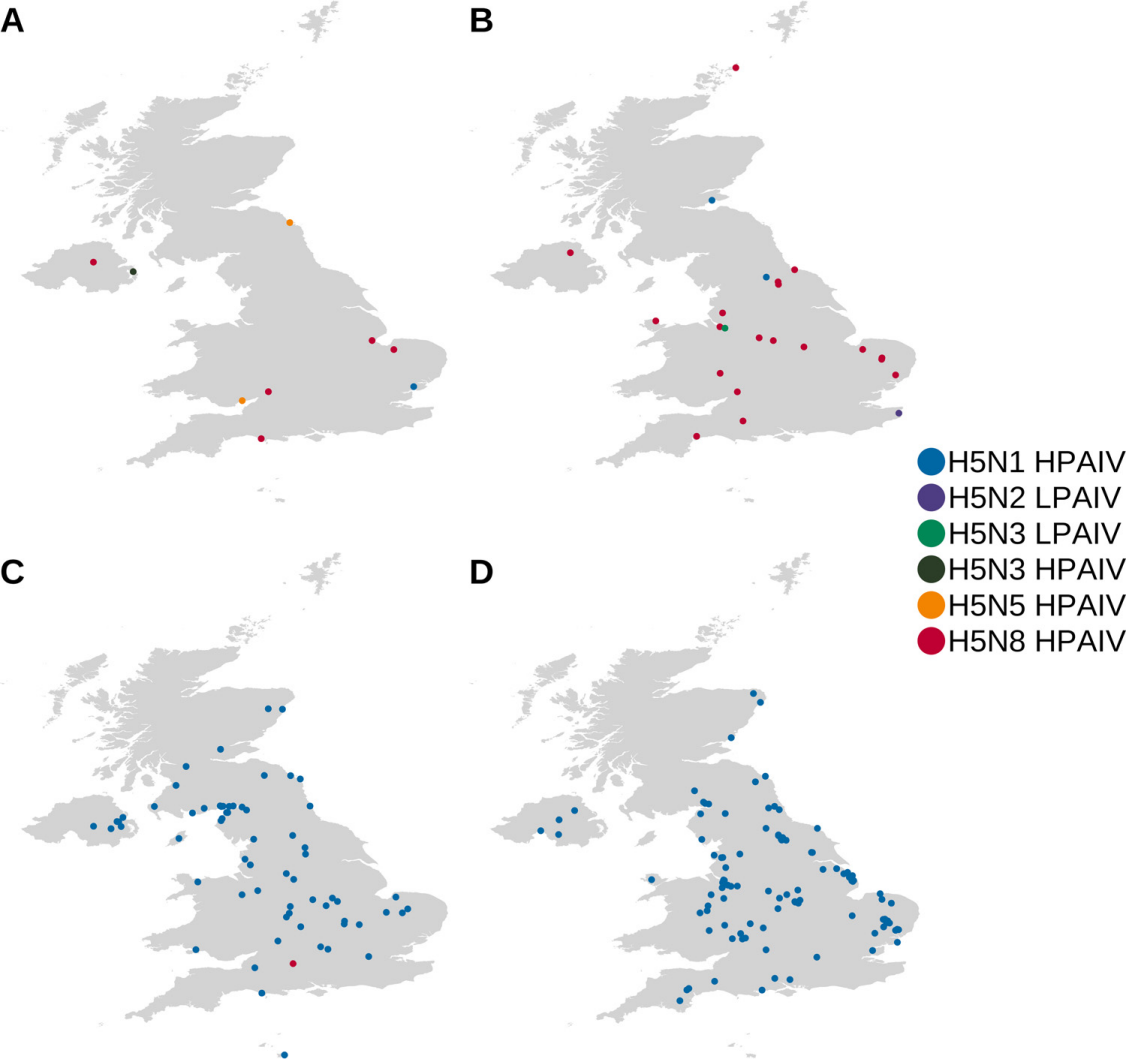
Geographic distribution of H5Nx AIVs that were sequenced during 2020 to 2022. Shown are the geographic distributions of H5Nx AIVs that were sequenced from wild birds (A and C) and poultry (B and D) during the 2020–2021 (A and B) and 2021–2022 (C and D) epizootics in the United Kingdom. Locations are colored according to the AIV subtype and pathotype.

**FIG 2:**
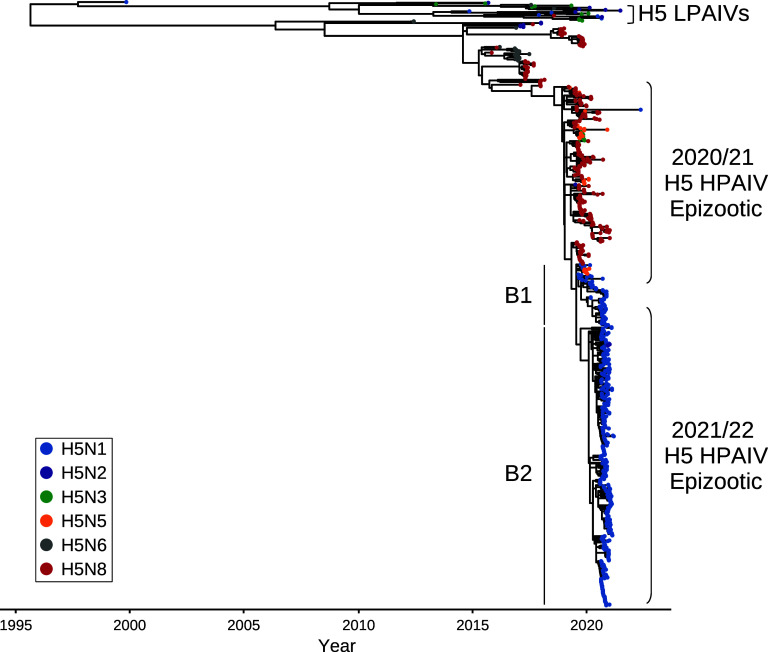
The HA of the H5Nx HPAIVs from 2020–2022 were derived from a common ancestor. Shown is a time-resolved maximum likelihood phylogenetic tree of the HA gene from H5Nx AIVs collected from the United Kingdom between 2020 and 2022, with relevant global reference sequences. The tips are colored according to AIV subtype, and the sequences obtained from either the 2020–2021 or the 2021–2022 H5 HPAIV epizootics are indicated. For the H5N1 HPAIV sequences, the B1 and B2 sublineages are also shown.

For H5N8 HPAIV detections in wild birds (Fig. 1A) and poultry (Fig. 1B) in the United Kingdom, WGS data demonstrated that all sequences were highly identical (>98.1%) across all gene segments, suggesting a single H5N8 genotype. Phylogenetic analysis of the United Kingdom H5N8 HA (Fig. 2; see also Fig. S1A in the supplemental material) and other gene segments (Fig. S1B to H) demonstrated high similarity to viruses detected in Europe during the same 2020–2021 epizootic period and implicated a single common H5N8 HPAIV ancestor, A/chicken/Iraq/1/2020, detected in May 2020. HPAIVs resulting from this ancestral strain were likely responsible for the spread across Europe as well as the Middle East and Central Asia. The HA cleavage site (CS) motif of the United Kingdom H5N8 sequences was PLREKRRKR/GLF, with only two sequences showing any differences (both P**Q**REKRRKR/GLF) (Table 1) (29).

**TABLE 1:** H5Nx subtypes and genotypes identified by WGS in the United Kingdom during 2020 to 2022

Subtype	No. of genotypes identified	Representative virus	Total no. of sequences	HA cleavage site motif(s)	Sector(s)
2020–2021 epizootic					
H5N1 HPAIV	1	A/chicken/England/043315/2020	3	PLREKRRKR/GLF	Poultry, wild birds
H5N2 LPAIV	1	A/environment/England/030642/2020	1	PQRETR/GLF	Poultry
H5N3 LPAIV	1	A/turkey/England/018179/2021	1	PQRETR/GLF	Poultry
H5N3 HPAIV	1	A/peregrine falcon/Northern Ireland/AI102021-2/2021	1	PLREKRRKR/GLF	Wild birds
H5N5 HPAIV	2	H5N5.1, A/Brent goose/England/095684/2020	1	PQREKRRKR/GLF	Wild birds
		H5N5.2, A/mute swan/Wales/048068/2020	2	PLREKRRKR/GLF	
H5N8 HPAIV	1	A/greylag goose/England/032698/2020	32	PLREKRRKR/GLF (*n* = 30), PQREKRRKR/GLF (*n* = 2)	Poultry, wild birds
2021–2022 epizootic					
H5N1 HPAIV	6	AIV07-B1, A/chicken/England/053052/2021	25	PLREKRRKR/GLF	Poultry, wild birds
		AIV07-B2, A/greylag goose/England/054503/2021	86	PLREKRRKR/GLF	Poultry, wild birds
		AIV08, A/chicken/Wales/053969/2021	2	PLREKRRKR/GLF	Poultry, wild birds
		AIV09, A/chicken/Scotland/054477/2021	82	PLREKRRKR/GLF (*n* = 78), PLKEKRRKR/GLF (*n* = 1), PLREKRKKR/GLF (*n* = 3)	Poultry, wild birds
		AIV20, A/turkey/England/016515/2022	1	PLREKRRKR/GLF	Poultry
		AIV55, A/chicken/England/069816/2021	1	PLREKRRKR/GLF	Poultry
H5N8 HPAIV	1	A/mute swan/England/298902/2021	1	PLREKRRKR/GLF	Wild Bird

**FIG 3:**
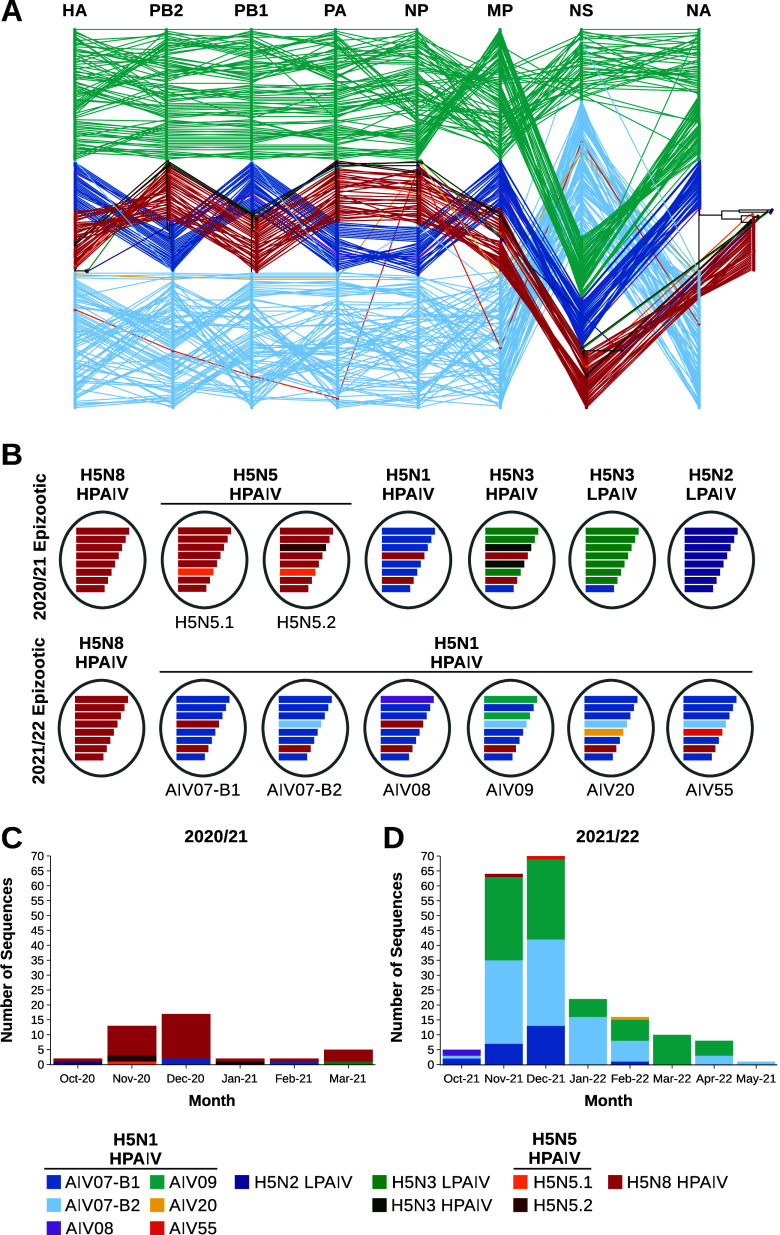
H5Nx AIVs from the United Kingdom collected between 2020 and 2022 demonstrate wide genotypic diversity. (A) Phylogenetic incongruence analysis of H5Nx sequences from the United Kingdom for AIVs collected between 2020 and 2022. Maximum likelihood phylogenetic trees for all gene segments from each strain are connected across the trees, with tips and connecting lines colored according to genotype. (B) Schematic representation of the different H5Nx genotypes from the United Kingdom between 2020 and 2022. Note that while the HA gene of the H5N1 HPAIV B2 sublineage is colored differently for the purposes of this diagram, it is still derived evolutionarily from the A/chicken/Iraq/1/2020 H5N8 HPAIV HA gene. (C and D) Numbers of sequences for each UK H5Nx genotype generated during the 2020–2021 (C) and 2021–2022 (D) epizootics.

H5N5 HPAIV was detected only in wild birds in the United Kingdom, and three genome sequences were generated from the samples collected (Table 1). However, even within this small number of sequences, two distinct H5N5 genotypes were identified by phylogenetic analysis. Both genotypes derived the majority of their gene segments from A/chicken/Iraq/1/2020, similar to the H5N8 HPAIV genotype observed in the United Kingdom (Fig. S1A to H), and shared the same two HA CS motifs (Table 1). The N5 gene appeared to be obtained through reassortment with local AIVs, as it demonstrated similarity to H5N5 AIVs detected in Europe in 2020 (Fig. S1B). However, the two United Kingdom H5N5 HPAIV genotypes differed in the PA gene. While H5N5.1, represented by A/Brent goose/England/095684/2020, detected in Northumberland (Fig. S2A), had a PA gene that was highly similar to that of A/chicken/Iraq/1/2020, H5N5.2, represented by A/mute swan/Wales/048068/2020, had a different PA segment closely related to those of AIVs detected in Eurasia, indicating potential reassortment (Fig. 3A and B and Fig. S1E). Nevertheless, this PA segment was also observed in H5N5 AIVs identified in European wild birds and poultry in 2020–2021.

The third HPAIV subtype detected during the 2020–2021 epizootic, H5N1, was identified only in England and Scotland. These H5N1 HPAIVs, like those also observed in Europe, demonstrated similarity in their HA (Fig. 2 and Fig. S1A) and matrix protein (MP) gene segments with A/chicken/Iraq/1/2020 H5N8 HPAIV (Fig. S1G). However, the other gene segments were highly identical to H5N1 sequences detected throughout Europe and Africa from 2020–2021, with relatedness to Eurasian AIV sequences from as far back as 2016, resulting in a singular genotype (Fig. 3A and B).

H5N3 HPAIV was detected in the United Kingdom in a single peregrine falcon (Falco peregrinus) from Northern Ireland (Table 1), being characterized as a reassortant, including the HA (Fig. 2 and Fig. S1A) and MP (Fig. S1G) genes from A/chicken/Iraq/1/2020 (Fig. 3A and B) and the remaining genes from Eurasian LPAIVs. Interestingly, the H5N3 HPAIV NS gene segment had >97% identity to the H5N1 HPAIV sequences.

The two LPAIVs were detected in England during 2020–2021 and included an H5N2 virus isolated from fecal material in Kent from a mixed-poultry premises and an H5N3 virus from turkeys in Cheshire. The sequence of the H5N2 LPAIV was genetically similar to other H5N2 sequences obtained from Europe and Asia during the same period (2020–2021) (Fig. S1A to H). The H5N3 LPAIV showed limited similarity to the other sequences obtained from the United Kingdom during this period, although the PB2, PB1, and NS segments clustered with those of the United Kingdom H5N3 HPAIV sequence.

### Reemergence and dominance of H5N1 HPAIV during the 2021–2022 season.

The reemergence of H5N1 HPAIV started following detection within the great skua (Stercorarius skua) population on the Shetland Islands off the northern coast of Scotland during the summer of 2021 (24) and was followed by further detections in the United Kingdom in wild birds and poultry from October 2021. The first poultry case occurred in Worcestershire on 26 October 2021, with the first wild bird detection being made in a gull (Larus canus) collected on 14 October 2021 from Scotland through the United Kingdom passive surveillance system. From these initial incursions, until May 2022, over 1,000 wild birds tested positive for H5N1 HPAIV across the United Kingdom, with a significant impact on the United Kingdom poultry sector, involving infections on over 115 premises. All poultry cases involved infection with the H5N1 virus that had circulated at a lower frequency during 2020–2021.

During the 2021–2022 epizootic, WGS of 196 viruses obtained from poultry and wild birds (Fig. 1C and D) in the United Kingdom demonstrated the presence of six distinct genotypes (Table 1). These genotypes were based on identity to the progenitor H5N1 HPAIV detected in the previous year (2020–2021) and found in the great skua population; genotypes are denoted based on the first detection in wild birds or poultry (Fig. 3A and B).

The first H5N1 genotype detected was AIV07, which had the same gene constellation as that of the H5N1 virus detected across wild birds and the two United Kingdom poultry cases during the previous epizootic (Fig. 3A and B and Fig. S1A to H). However, this genotype initially demonstrated divergence within the HA gene (23) (Fig. 2) and has since been defined as two separate genotypes. The AIV07-B1 genotype contained an HA with high similarity to that of the H5N1 HPAIV from 2020–2021 and was the primary United Kingdom H5N1 virus detected during 2021 to 2022. However, AIV07-B1 later became a minority population in the United Kingdom and was not detected after February 2022 (Fig. 3C). The AIV07-B2 genotype possessed an HA gene that had diverged from that of AIV07-B1 (23) (Fig. 3C), although both genotypes were detected in wild bird and poultry cases throughout the United Kingdom (Fig. S2C and D).

The third H5N1 genotype, AIV08, was detected in only a single poultry case and an associated wild bird detection from the same site in Wales in October 2021 (Fig. S2C and D). The AIV08 genotype showed high genetic similarity in all gene segments to AIV07-B1, except for the PB2 segment (Fig. 3A and B), which had high similarity to that observed in LPAIVs detected in the Netherlands and the Republic of Ireland since 2018 (Fig. S1C). H5N1 HPAIV sequences with similar PB2 segments were detected in poultry and wild birds in France, Italy, Moldova, and Romania between October 2021 and February 2022.

The fourth H5N1 genotype, AIV09, was the second most prevalent genotype (Table 1 and Fig. 3C) and was first detected in Scotland in November 2021 but has since been detected across the United Kingdom in poultry and wild birds (Fig. S2C and D). The AIV09 genotype shared the PB1, NP, NA, MP, and NS segments with AIV07-B1 and AIV07-B2 but possessed the HA from the B2 sublineage. The PB2 and PA segments, however, demonstrated dissimilarity to both AIV07 genotypes as well as AIV08 (Fig. 3A and B). The AIV09 gene showed high genetic similarity to those seen in the H5N3 AIVs in the United Kingdom and Europe during the 2020–2021 epizootic (Fig. S1C), the PA showed high genetic similarity to LPAIVs from the Netherlands and Belgium detected since 2017 (Fig. S1E). Interestingly, phylogenetic incongruence analysis suggests that there may be two separate lineages within the AIV09 genotype based on differences in the NS segment (Fig. 3A). However, the nucleotide identity of all of the United Kingdom H5N1 sequences from 2020 to 2022 share >98.21% identity in the NS gene, and the topography of the phylogenetic tree demonstrates that the European H5N1 NS genes were derived from a single common ancestor (Fig. S1H). Therefore, it can be inferred that the NS gene is the same across the AIV09 genotype sequences.

The final two H5N1 HPAIV genotypes, AIV20 and AIV55, were detected only once during the study period (October 2020 to May 2022). AIV20 was detected on a turkey farm in Lincolnshire in February 2022, while AIV55 was detected in chickens from County Durham in December 2021 (Fig. 3C and Fig. S2D). Both genotypes shared seven of their eight gene segments with AIV07-B2, including the HA gene, but had alternative NP gene segments (Fig. 3A and B). For AIV20, the NP segment demonstrated similarity to those from AIVs in the Netherlands and Belgium but also to those from the H5N3 HPAIVs observed during 2020 to 2021 (Fig. S1F). The AIV55 NP segment was more closely related to those observed in H5N1 and H5N5 HPAIVs from Eastern Europe and Russia as well as an H12N5 sequence from Belgium.

Finally, while no detections of H5N8 HPAIV were made in poultry during the 2021–2022 season, this subtype was found in a single mute swan (Cygnus olor) from Wiltshire in November 2021 (Fig. 1C). The virus sequence demonstrated high similarity to the H5N8 virus observed in the United Kingdom during the 2020–2021 epizootic and was of the same genotype (Fig. 3A and B and Fig. S1A to H).

### Evaluation of host tropism markers in H5Nx sequences.

According to standard risk assessments in the United Kingdom, AIV sequences obtained from outbreaks were assessed for the presence of previously defined zoonotic molecular markers associated with increased virulence, alterations in host tropism, and resistance to antivirals (30, 31) (Table S2). A number of polymorphisms were identified, including the HA T156A substitution, which is associated with increased binding to α2-6-linked sialic acids (31–33). Interestingly, the PB1 D3V substitution was identified within the majority of the sequences and genotypes assessed but was differentially identified between the two H5N5 genotypes; this substitution was present in the H5N5.1 genotype, while it was absent from H5N5.2. This may indicate genetic drift between the two genotypes, but this cannot be confirmed given the limited number of H5N5 HPAIV sequences obtained. The H5N5 and H5N8 sequences were also exclusively found to possess a truncated PB1-F2 protein, consisting of only 11 amino acids, while all other sequences had a full-length (90-amino-acid) protein.

The M2 A30S amino acid substitution, associated with reduced susceptibility to amantadine and rimantadine (31, 34–38), was identified in a single H5N1 sequence, while the NA I117T substitution, shown to reduce susceptibility to NA inhibitors (31, 39), was identified in all H5N2 and H5N3 sequences. The PA I38T substitution, associated with reduced baloxavir susceptibility (40), was not identified in any of the sequences analyzed.

### Genetic assessment of outbreak clusters to assess the likelihood of lateral spread.

**FIG 4:**
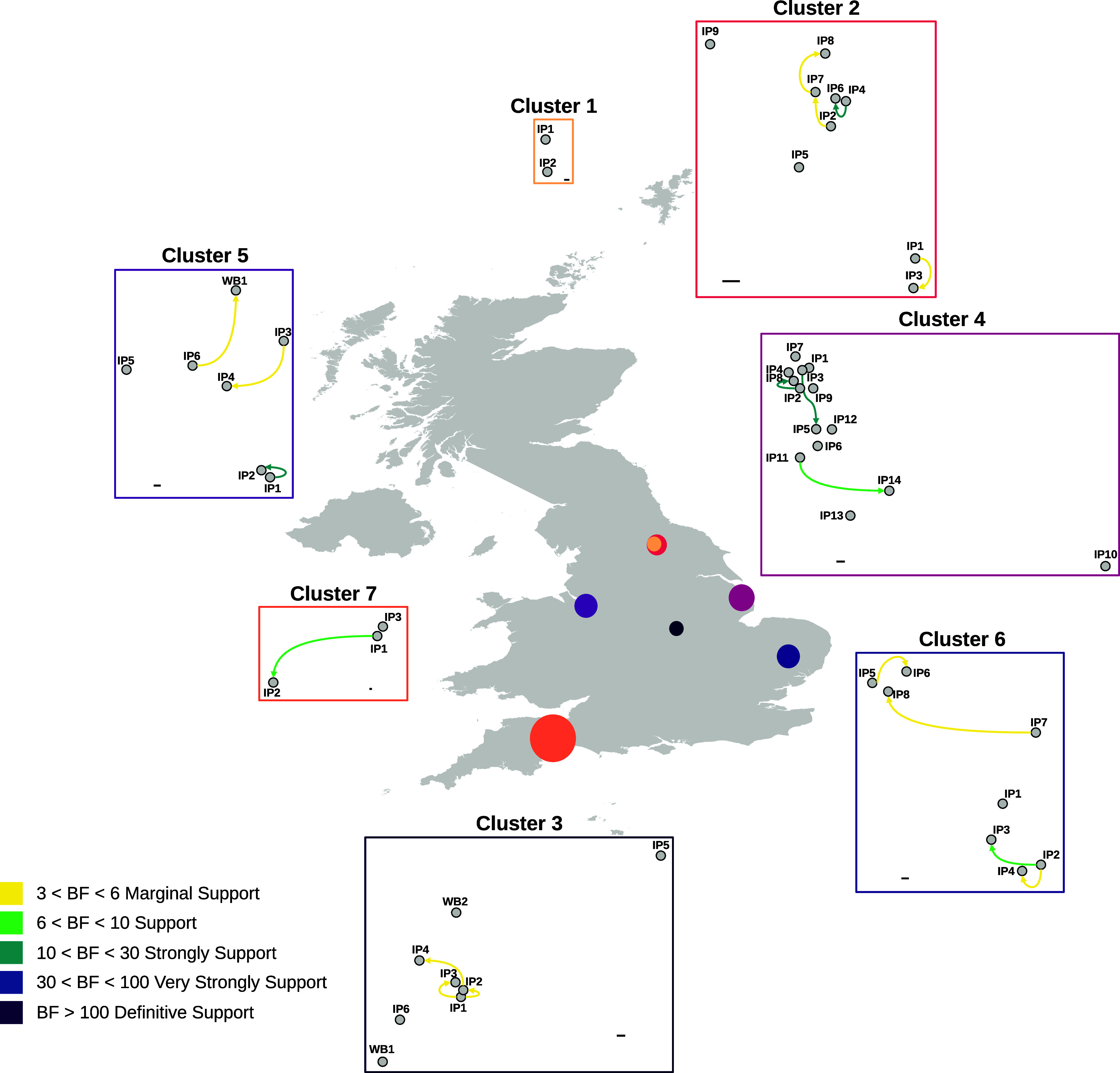
Analysis of H5Nx sequences suggests limited lateral transmission between geographically related HPAIV detections. Shown are outputs of the BSSVS analysis for the seven geographic clusters of H5Nx HPAIV detections investigated for the potential of lateral transmission to have occurred. Each geographic cluster is represented by a separate network diagram using the relative location of each infected premises (IP) or wild bird (WB) detection. Arrows are colored according to the relative strength, inferred using a Bayes factor (BF), by which the transmission rates are supported. Scale bars are provided for each cluster, representing 1 km.

During the 2020–2021 epizootic, the H5N8 sequences characterized shared high genetic identity and formed a single genotype. The geographic distribution of cases in the United Kingdom during 2020–2021 suggested that there was no direct epidemiological relationship between infected premises (IPs) and supports the likelihood of multiple independent primary introductions from wild birds in each instance. The movement of birds prior to the development of disease may, of course, have facilitated outbreaks in geographically distinct areas, but evidence to evaluate this could not be drawn from genetic data. One exception to this was two closely linked IPs located in North Yorkshire (cluster 1), which were confirmed to be H5N8 HPAIV positive within 4 days of each other, and epidemiologically defined pathways were demonstrated (data not shown) (Table S3). A Bayesian stochastic search variable selection (BSSVS) analysis was used to identify well-supported rates of transition between IPs, and support was quantified using Bayes factors (BFs) (Fig. 4 and Table S4). This analysis demonstrated that there was no strong BF support for transmission between premises, suggesting separate introductions at both premises.

The escalation of cases during the 2021–2022 epizootic led to further investigations into the potential for lateral spread between IPs. Six geographically linked groups of premises, or “clusters,” were detected within short time frames of each other in poultry-dense regions of England (41). The presence of multiple H5N1 HPAIV genotypes in circulation within the United Kingdom enabled the distinction of independent incursions wherever different genotypes were detected. This also enabled the refinement of the IPs that constituted each cluster based on the major genotype detected. A BSSVS analysis approach was then applied to this refined set of IPs to assess the potential for lateral spread as opposed to independent introductions. Of the six clusters investigated from the 2021–2022 epizootic, all but one involved the AIV09 genotype, with only cluster 5 involving the AIV07-B2 genotype (Table S3).

Cluster 2 consisted of nine IPs where AIV was detected between 12 November and 8 December 2021, including a mixture of chicken and turkey premises. The BSSVS analysis suggested the potential for lateral spread between IP4 and IP6 (both turkey premises), with strong BF support (Fig. 4 and Table S5).

Cluster 3 involved six IPs (five chicken and one turkey premises), and two wild bird detections, a mute swan and a common gull, that were collected between 13 November 2021 and 8 January 2022 in Leicestershire. Interestingly, using this approach, neither wild bird sequence was proposed to be the origin of H5N1 for the poultry premises within this cluster. BSSVS analysis suggested that IP1 was a potential source of virus for both IP2 and IP3, with the former also being linked to IP4. Furthermore, these IPs were all chicken premises located in proximity to each other. The BF support for lateral transmission was low for all other IPs in this cluster, suggesting independent introductions from wild birds directly or indirectly (Fig. 4 and Table S6).

Cluster 4 was the largest geographic cluster investigated, involving 14 IPs, chickens (*n* = 10), ducks (*n* = 1), turkeys (*n* = 2), and 1 IP housing chickens and ducks (IP12), that were detected between 11 December 2021 and 8 January 2022 in Lincolnshire. However, there appeared to be only two strongly supported transmission events following BSSVS analysis, between IP3 and IP5 and between IP2 and IP8, all four of which were chicken premises. Interestingly, IP2 and IP8 were located close to each other, but IP3 and IP5 were more distant, with several other premises being situated between them in the line of flight. The BSSVS analysis suggested that the remaining IPs were likely the result of independent introductions (Fig. 4 and Table S7).

Cluster 5 involved six poultry IPs (one chicken, one duck, and four turkey premises) and a wild bird detection (a mute swan) confirmed between 17 December 2021 and 27 January 2022 in Cheshire. Within this cluster, transmission from IP3 to IP4 (both turkey premises) had marginal support, as did transmission from IP6 to wild bird 1 (WB1) (Fig. 4 and Table S8). However, given that WB1 was detected over a month before IP6 was detected (Table S3), this may indicate that both detections were the result of a singular, unidentified introduction source, most likely another wild bird. Only the transmission between IP1 and IP2, which were in proximity to each other, was strongly supported by the BSSVS analysis.

Cluster 6 consisted of eight IPs (one goose, five duck, and two chicken premises) confirmed between 25 February and 4 April 2022 in Suffolk. The BSSVS analysis suggested transmission from IP2 to IP3 and IP4 as well as transmissions from IP5 to IP6 and from IP7 to IP8 (Fig. 4 and Table S9). However, these transmissions were supported by low BFs, with infections at the remaining premises being proposed to be independent introductions.

Cluster 7 was the last cluster to be identified and investigated, consisting of three premises (one containing both ducks and geese along with one duck and one chicken premises) detected between 4 and 12 April 2022 in Devon. The BSSVS analysis suggested that IP1 may have transmitted the virus to IP2, which were the most distant IPs geographically; however, the support for this was low (Fig. 4 and Table S10).

## DISCUSSION

The 2020–2021 European HPAIV epizootic involved 3,555 virus detections across 28 countries (20). While H5N8 predominated during that epizootic (88% of the total detections), multiple other subtypes were detected, including H5N5 in captive birds, poultry, and wild birds (3% of the total detections); H5N1 in poultry and wild birds (3% of the total detections); as well as H5N3 and H5N4 in wild birds (1% and 0.5% of the total detections, respectively), with the majority being detected between 5 October 2020 and 23 February 2021 (20). In the United Kingdom, there were totals of 26 positive poultry premises and 320 positive wild birds, with the majority of cases being H5N8, followed by H5N1, H5N5, and H5N3. H5N4 HPAIV, while detected in Germany, the Netherlands, and Switzerland, was not detected in the United Kingdom (20). The high degree of genetic relatedness to A/chicken/Iraq/1/2020 across H5 HPAIV subtypes supports the hypothesis that H5N8 was introduced into Europe via a single common progenitor, most likely during late 2020 via Russia and Eastern Europe (42), although the migratory movements driving emergence remain undefined. Regardless of the mechanism of introduction, multiple H5 HPAIV subtypes with significant genotypic diversity were detected (43). Critically, while reassortants involving some internal genes have been described, the HA and MP gene segments were conserved throughout the European H5 HPAIV detections in 2020–2021 (43). In the United Kingdom, genotypic diversity was limited to a single H5N8 and two H5N5 genotypes during the 2020–2021 epizootic. While this homogeneity in United Kingdom genotypes is difficult to explain, it may be a result of the rapid lockdown of infected premises reducing the risk of onward transmission and further genetic diversification.

During 2020–2021, two H5 LPAIVs (H5N2 and H5N3) were detected in unrelated poultry cases in the United Kingdom. From a genetic standpoint, the H5N3 LPAIV contained three gene segments with high sequence identity to the H5N3 HPAIV detected in Northern Ireland in January 2021. The detection of notifiable LPAIVs often relies on serological flock assessments and rapid statutory follow-up investigations of premises where H5- or H7-specific antibodies are detected. Both of these detections were the result of this testing algorithm, and neither of the LPAIVs detected in these instances caused any overt clinical disease in the birds involved. The paucity of data underscores our lack of understanding of LPAIV circulation. However, having the environment for interactions between species that might transmit both LPAIVs and HPAIVs is critical for coinfection events and the factors, including susceptibility and prior immunity, that drive this.

The last detection of H5N1 HPAIV in poultry in the United Kingdom for the 2020–2021 epizootic was in late March 2021. In contrast, the detection of HPAIV across Northern and Eastern Europe in poultry and wild and captive birds continued to May 2021 (20). For the first time, summer detections of H5N1 HPAIV occurred following emergence in great skuas off the northern coast of Scotland during July and August 2021 (24), with the virus being closely related to the H5N1 HPAIV detected in the United Kingdom and Europe during the 2020–2021 epizootic. This virus was also detected a further 54 times during the summer of 2021 in wild birds from Europe (Estonia, Germany, Finland, Latvia, the Netherlands, and Sweden) (21), suggesting the maintenance of this virus across wild bird populations (24).

Within the United Kingdom, two sublineages of the AIV07 genotype, AIV07-B1 and AIV07-B2, were detected between October 2021 and May 2022 in 12 poultry IPs and 10 wild birds and in 42 poultry cases and 44 positive wild birds, respectively. The AIV07-B1 genotype was also detected in the human case of H5N1 HPAIV infection during December 2021, although no evidence of mammalian adaptation was observed (44). While the H5N1 B1 sublineage has been detected across North America since the end of the 2020–2021 European HPAIV epizootic (25, 26), it was a minor sublineage detected in Europe during the 2021–2022 epizootic (United Kingdom, *n* = 25; France, *n* = 1; Germany, *n* = 7; Republic of Ireland, *n* = 5; Sweden, *n* = 7; Denmark, *n* = 1). The reemergence of HPAIV in the United Kingdom is hypothesized to have occurred via two routes: (i) the AIV07-B1 genotype was likely introduced from Sweden and Denmark, while (ii) the AIV07-B2 genotype was likely introduced from Northern Europe, having likely originated in Russia and Eastern Europe. In both cases, the virus was likely introduced following the movements of migratory waterfowl, although local asymptomatic circulation in local wild bird populations cannot be excluded.

The detection of the AIV08 genotype in only a single poultry case and a single associated wild bird case during the 2021–2022 season is of interest. The clustering of the AIV08 HA with the H5N1 B1 sublineage suggests potential emergence following reassortment between an AIV07-B1 virus and an undefined AIV present within wild birds. Its apparent extinction in the United Kingdom and the limited detection of AIVs containing a PB2 segment similar to this genotype across Europe may indicate poor segment compatibility, perhaps resulting in reduced viral fitness or a different host tropism.

The third genotype detected within the United Kingdom, AIV09, has high sequence identities to the AIV07 genotypes but contains different PB2 and PA genes. The B2 sublineage HA of AIV09 suggests that this reassortment event occurred following the emergence of the divergent HA gene. It is hypothesized to have been introduced into the United Kingdom from the East due to its relatedness to contemporary H5N1 viruses detected in late summer in Russia.

The AIV20 and AIV55 genotypes showed substantial similarity to the AIV07-B2 genotype, except for their NP genes. These genotypes may have followed a migration pathway similar to that of the AIV07-B2 genotype but potentially obtained their novel NP genes through reassortment with AIVs circulating in European wild birds.

An assessment of the sequences generated in this study for polymorphisms associated with increased virulence, altered host tropism, or antiviral resistance found that there was no association between the H5 genotype and the observed polymorphisms. Previous studies have demonstrated that adaptive changes occur within the polymerase complex following mammalian infection but that the change identified (PB2 D701N) was most likely a single mutation that, alongside other mammalian adaptations, may increase the zoonotic threat (9). Similarly, PB2 E627K, shown to be involved in adaptation to mammalian hosts (31, 45–49) and considered a significant marker of mammalian adaptation, was identified in only a single H5N1 sequence obtained from United Kingdom poultry. Nevertheless, the risk of infection at the poultry-human interface posed by these H5Nx clade 2.3.4.4b viruses remains low, as evidenced by the low number of human infections that have been detected globally since 2020 (10, 50) despite the substantial infection pressure and potential for opportunistic infections at the avian-human interface during the concurrent epizootics.

The apparent maintenance of H5N1 within wild bird species during the summer months of 2021 in Northern Europe is a key shift in epidemiology compared to what has been previously observed for clade 2.3.4.4b H5 HPAIVs. The apparent stability of the different H5N1 genotypes following introduction into the United Kingdom in late 2021 is clearly demonstrated by the genotype distribution across overlapping locations and disparate species. As described above, defining the origin of these genotypes is problematic without a greater understanding of the circulation of HPAIVs in species that tolerate infection in the absence of disease and LPAIVs among all bird species.

The genetic analysis of different viruses from geographically linked clusters aimed to define where independent incursions may have occurred over the likelihood of lateral spread due to inefficient biosecurity practices. The determination of multiple H5N1 genotypes during the 2021–2022 outbreak enabled, at least where different genotypes were observed, some conclusions to be made at the consensus level, although genetic divergence could not conclusively be used to differentiate between introduction sources, and a more rigorous approach was required (51–54). Investigations of this type will become more important for understanding incursion risks and the factors driving virus spread. Certainly, the utility of WGS in characterizing outbreaks is critical, and comprehensive genetic data, particularly for LPAIVs, with a deeper level of analysis would benefit assessments of this type.

In conclusion, the changes in HPAIV epidemiology and maintenance within local populations raise uncertainties in defining the risk of incursions. Interestingly, the two United Kingdom epizootic events appear to have demonstrated differential plasticity in HA/NA interactions, with the 2020–2021 H5 successfully interacting with multiple NA types but the 2021–2022 H5 exhibiting an apparent preferential interaction with N1 that has facilitated proliferation across a broader range of species than previously observed. Furthermore, the replication fitness of these viruses appears to have a tolerance for the reassortment of several segments, particularly the polymerase complex. A rapid evaluation of factors influencing the impact of genotype on phenotype is required to better understand virus-host interactions.

## MATERIALS AND METHODS

### Whole-genome sequencing.

United Kingdom regulations categorize H5 AIVs as Specified Animal Pathogens Order 4 (SAPO4) notifiable animal disease agents (55); as such, all laboratory work with H5 AIV samples was performed in licensed biosafety level 3 (BSL3) facilities. The samples (oropharyngeal or cloacal swab fluids, or tissue homogenates: brain, lung, trachea, instestines or mixed viscera) obtained from H5 AIV-positive investigations identified by real-time reverse transcription-PCR (RT-PCR) (56) or virus isolates derived from these samples were used to generate whole-genome sequences. Virus isolates were obtained from clinical samples using 9- to 11-day-old specified-pathogen-free embryonated fowls’ eggs (2). Total RNA was manually extracted from either clinical samples or viral isolates, without the addition of carrier RNA (57).

The extracted RNA was converted to cDNA using the SuperScript IV first-strand synthesis system with random hexamers (Thermo Fisher) and then to double-stranded cDNA using the NEBNext Ultra II nondirectional RNA second-strand synthesis module (New England BioLabs). The double-stranded cDNA was then purified and concentrated using Agencourt AMPure XP beads (Beckman Coulter), incubated at room temperature for 5 min, and eluted in 10 μL of 1 M Tris-HCl (pH 7.5) (Sigma) before quantification using the QuantiFluor double-stranded DNA (dsDNA) system (Promega). For the preparation of the sequencing library, 1 ng of purified dsDNA was used as the template, and the library was generated using the NexteraXT kit (Illumina). Sequencing libraries were run on either a MiSeq or a NextSeq 550 platform (Illumina) with 2× 150-bp paired-end reads.

Raw sequencing reads were assembled using custom scripts: either FluSeqID (https://github.com/ellisrichardj/FluSeqID) with a consensus sequence generated using genconsensus.py (https://github.com/AMPByrne/WGS/blob/master/genconsensus.py) or denovoAssembly (https://github.com/AMPByrne/WGS/blob/master/denovoAssembly_Public.sh). Some of the sequences used in this study were used in previous studies (9, 23, 44), but all sequences produced as part of this study are available through the GISAID EpiFlu Database (https://www.gisaid.org/) (see Table S1 in the supplemental material).

### Phylogenetic analysis.

Given the diverse nature of the H5Nx subtypes and genotypes observed in Europe during 2020 to 2022, it was important that an appropriate phylogenetic reference data set be assembled to maintain the resolution of any subsequent analysis. To achieve this, all global AIV sequences from 2014 to 2022 were obtained from the GISAID EpiFlu Database and combined with the United Kingdom sequence data described in this study. This combined data set was then used to generate phylogenies for each influenza virus gene segment using Nextstrain (58), and any duplicate sequences, sequences of poor quality, or sequences demonstrating no topological relatedness to the United Kingdom sequences of interest were manually removed to provide the final sequence data set.

The sequences of the final data set were then aligned for each gene using Mafft v7.487 (59) and manually trimmed to the open reading frame using AliView (60). Phylogenetic trees were then inferred using the maximum likelihood approach in IQ-Tree v2.1.4 (61) with ModelFinder (62) to infer the appropriate phylogenetic model and 1,000 ultrafast bootstraps (63). Ancestral sequence reconstruction and inference of molecular-clock phylogenies were performed using TreeTime (64). Phylogenetic trees were visualized using R version 4.1.1 with the libraries ggplot2, ggtree (65), and treeio (66). Phylogenetic incongruence analysis was performed using the maximum likelihood phylogenetic trees with the backronymed adaptable lightweight tree import code (BALTIC) as described previously (67). Graphs were generated using Plotly version 5.8.0 (Plotly Technologies Inc.).

### Evaluation of viral polymorphisms associated with altered AIV characteristics.

Viral protein sequences were screened for the presence of genetic polymorphisms that had been previously demonstrated to be associated with altered viral virulence, host tropism, and antiviral resistance (30, 31) using a custom script, https://github.com/dombyrne/Influenza-Mutation-Checker, and database, which is available upon request.

### Cluster analysis.

An assessment of the potential for lateral spread versus independent introduction was undertaken on geographically linked cases, termed clusters. For each cluster, all wild bird and poultry detections that were geographically and temporally relevant and for which whole-genome sequencing (WGS) data had been obtained were included. Given that the different H5Nx genotypes were distinct, only sequences that were of the predominant genotype within the cluster sequences were included. These sequences were then concatenated to generate a single full-genome sequence covering all genes for each detection. The concatenated sequences were combined with a concatenated version of the genotype reference sequence (Table 1) and aligned as described above.

Time-scaled phylogenetic trees were then inferred from the aligned concatenated sequences using BEAST version 1.10.4 (68) with the BEAGLE library (69). The Shapiro-Rambaut-Drummon-2006 (SRD06) nucleotide substitution model was implemented with a four-category gamma distribution model of site-specific rate variation and separate partitions for codon positions 1 plus 2 versus position 3 with the Hasegawa-Kishino-Yano (HKY) HKY substitution models on each with an uncorrelated relaxed clock with and log-normal distribution and the coalescent constant population size tree prior. For each cluster data set, two independent Markov Chain Monte Carlo (MCMC) chains were run and combined using the LogCombiner tool in the BEAST package. Each chain consisted of 200,000,000 steps and was sampled every 20,000 steps, and the first 10% of samples were discarded as the burn-in. The MCMC settings were chosen to achieve a post-burn-in effective sample size of at least 200. Discrete transition events between cluster detections were reconstructed using a symmetric continuous-time Markov Chain model with an incorporated Bayesian stochastic search variable selection (BSSVS) to determine which transition rates sufficiently summarized the connectivity between detections (70). SpreaD3 was used to visualize the rates of transmission using a Bayes factor (BF) test (71). The BF represents the ratio of two competing statistical models, represented by their marginal likelihood, and, in this case, was used to determine the likelihood of transmission between detection events, as opposed to independent introductions (72). The support of the BF for transmission was interpreted as described previously (73). Within each cluster, transmission events with a supporting BF value of <3 or with a supporting BF value lower than that between any of the cluster sequences and the reference sequence, whichever was higher, were omitted.

### Ethics statement.

All samples were obtained from dead animals collected as part of the epizootic.

### Data availability.

All sequence data generated and used in this study are freely available through the GISAID EpiFlu Database (https://www.gisaid.org/). All GISAID accession numbers are provided in Table S1 in the supplemental material.
